# Hybrid approach: a prospective option for treating congenital heart defects in pediatric patients

**DOI:** 10.3389/fcvm.2025.1711494

**Published:** 2025-12-16

**Authors:** Shukhrat Marassulov, Oleg Lookin, Bakhytzhan Nurkeyev, Amangeldy Kerimkulov, Saniya Murzabayeva, Bauyrzhan Tuyakbayev, Raikhan Dochshanova, Rinat Maiorov, Assiya Akhmoldayeva, Elmira Kuandykova, Yerbol Aldabergenov, Timur Raimkhanov, Akkerbez Adilbekova

**Affiliations:** JSC National Scientific Medical Center, Astana, Kazakhstan

**Keywords:** congenital heart disease, ventricular septal defect, pediatric patients, hybrid approach, hybrid surgery, mini-invasive surgery

## Abstract

Congenital heart defects (CHDs), a life-threatening congenital pathology, are reported in approximately one out of every 100 live births, with the severity ranging from mild to fatal. The prevalence of CHDs has significantly increased over the last few decades, most likely due to evolved diagnostics and increased accessibility to healthcare worldwide. The ratio of severe CHDs, which require urgent surgery, to mild forms, which may not require surgery, is between 1:4 and 1:3. Therefore, every fourth or fifth newborn with a CHD needs immediate and effective surgical treatment. Furthermore, one in 10 diagnoses involves multiple CHDs, which require complex surgical treatment and elevate the risk of peri- and post-operative mortality. In this review, we focus on ventricular septal defects (VSDs) that constitute a significant proportion of CHDs. We briefly discuss the historical background and current strategies for VSD treatment, including open-heart surgery, transcatheter surgery, and mini-invasive hybrid surgery. The hybrid method is then comprehensively discussed, considering its success and complication rates compared to the other two approaches, its implementation, typical delivery approaches, and the most common types of occluders; we accompany this discussion with our own clinical experiences. The advantages and limitations of the hybrid approach are also discussed. We conclude that the prospects for wider use of the hybrid approach for VSD correction are favorable due to its mini-invasiveness, high safety and effectiveness, and because cardiopulmonary bypass is not needed in this approach.

## 1 Introduction

Congenital heart diseases (or congenital heart defects, CHDs) encompass a wide spectrum of cardiac defects that develop in the prenatal period and are present at birth. CHDs are one of the leading causes of birth defects, resulting in high morbidity, mortality, and healthcare costs. The most common types of CHD include defects in the ventricular or atrial septum, valves, and main vessels, and nearly 1 in 10 CHD diagnoses involve multiple CHDs (1–4). Such a high proportion of CHDs requires urgent surgical correction, and, if untreated, these CHDs are life-threatening and often fatal. Moreover, the severity of CHD directly contributes to pre-, peri-, and post-operative mortality because a severe CHD is often accompanied by other pathologies and complications, many of which are also congenital. Also, nine out of ten babies born with CHD worldwide are in areas with limited or no access to care, where mortality remains high (5). The wide spectrum of CHD brings a great economic and social burden. For example, the cost of hospitalizations for individuals with CHDs in the U.S. alone exceeded USD 9.8 billion in 2019 (6), highlighting the significant economic burden associated with CHD care. Therefore, it is clear that safe and robust surgical treatments for CHD are urgently needed.

The global diagnosis rate for various CHDs at birth is often reported to be between 8 and 10 per 1,000 live births—this value has been stable over the last three decades (7–11). CHDs are affecting many people; globally, about 12 million people live with CHDs, accounting for about 600,000 years of disability (5, 12). The reported values vary widely, e.g., from 2.5 to 50 per 1,000 live births, depending on the geographical region and the range of years selected for evaluation (13–37). The availability of proper diagnostic tools, the existence of nationwide prenatal screening programs, the severity of CHD (e.g., some studies report only severe CHD cases, while others report mild and severe CHD cases together), and other factors contribute to the significant variability in the reported CHD-related early mortality rates. This also includes both temporal variation, mostly explained by recent advancements in diagnosis, and geographical variation, mostly explained by regional discrepancies in healthcare systems. Also, other factors, such as race, ethnicity, socio-economic status, and ecology, may also contribute to the high variability in the reported prevalence.

Congenital cardiac defects at birth are life-threatening. The mortality risk in infants with severe forms of CHD during the first year of life is 12 times higher vs. those with mild forms of CHD (37). The risk for another critical complication, heart failure, is ∼6 times higher in these patients, and it develops in every 1 out of 13 adult survivors with CHD (38, 39). In general, about 20%–25% of CHDs are considered seriously life-threatening conditions and therefore require urgent surgical treatment (34, 40, 41). Additionally, nearly 10% of CHDs include multiple pathologies (42). In adulthood, some conditions like Eisenmenger syndrome, which results from longstanding unrepaired congenital heart defects, can cause significant morbidity and can be life-threatening (43). Moreover, CHDs have also been linked to “essentially non-cardiac” diseases, elevating the risk of dementia, CNS infections (44, 45) and type 2 diabetes mellitus (46) in individuals with severe CHD. Also, CHD-affected newborns have accompanying defects in the CNS and gastrointestinal and urogenital organs (24). This underscores the importance of early detection and management of CHD, especially for defects that pose immediate risks.

CHD-associated mortality remains high according to available reports worldwide, but the reported values greatly differ between studies. In neonates and during the first 5 years of life, the reported mortality rate varies significantly: 4% in the U.S (33).; approximately 10% in European, Central Asian, and South American countries (43, 47–49); as high rates as 15% in Malaysia (50); 17.4% in Saudi Arabia (25); and 30% in China (18). A recent study that analyzed data provided by the European Congenital Heart Surgeons Association Congenital Cardiac Database (ECHSA-CCDB) for the period 1999–2024 revealed a 10.28% in-hospital mortality rate in neonates with CHD (51). In premature neonates with CHD, the mortality rate is much higher, and 1-year survival is substantially lower compared to the normal-term neonates with CHD (52–55). A large study collected preterm birth clinical data between 2007 and 2015 from 10 countries (including Australia, Canada, Japan, and several European countries) and similarly revealed that the mortality rate in neonates with CHD is two-fold more frequent compared to those without CHD (56). On the other hand, some reports indicate that the mortality rate in neonates with diagnosed CHD may not differ between preterm and normal-term live births, regardless of whether the compared groups had moderate or severe CHD (57). Nonetheless, despite the overall positive global trends in CHD-related mortality across all age groups and children under 5 years, untreated CHD remains a critical challenge, significantly contributing to overall mortality (58).

The etiology of CHD is complex and multifactorial. Although the exact cause remains unknown in many cases, established risk factors include chromosomal and genetic abnormalities (e.g., trisomy 21 or Down syndrome), monogenic syndromes, maternal infections (e.g., rubella), pregestational diabetes, teratogenic drugs, alcohol, and other environmental exposures during pregnancy (59–62). For example, the association between congenital cardiac defects and Down syndrome, trisomy 13, or trisomy 18 is generally reported to be between 50% and 80% (63–65). About one-third to 40% of CHD cases are related to genetic variants, and the number of new such variants has constantly increased (66, 67). The inherited predisposition to CHD is a significant contributing factor because parents with CHD have a substantially increased risk of giving birth to offspring with CHD, and mothers have a two times higher risk compared to fathers (68). Because of the high proportion of genetically conditioned CHDs, genetic screening and testing in the prenatal stage is now considered one of the major contributors to survival (65), along with constant improvements in surgical approaches.

Also, an unequal prevalence of CHD in male and female neonates has been reported in several studies (62, 69, 70), while other studies did not reveal substantial sex differences (71, 72). It is interesting that the greater overall frequency of CHD and the prevalence of moderate CHD are both higher in female neonates, while the prevalence of severe CHD is higher in male neonates (23, 70, 73).

The rate of CHD detection (including ventricular septal defects, VSDs) has increased steadily over time, but this is unlikely to reflect greater fetal susceptibility to pathological remodeling. Rather, advances in various diagnostic tools and the implementation of local and national early screening programs for prenatal development defects have contributed to the rapidly increasing number of CHD diagnoses. Indeed, numerous studies have reported a dramatic increase in the diagnostic rate of major CHDs in the prenatal period and in neonates (16, 74, 75). Both timely diagnosis and proper surgical treatment of these defects are essential to prevent complications such as pulmonary hypertension, heart failure, arrhythmias, or irreversible myocardial damage.

In this review, we focus solely on ventricular septal defects (VSDs) that constitute a significant proportion of CHDs. The first section briefly summarize current point of view on VSD types and epidemiology. Currently most used strategies for VSD treatment—open-heart surgery, transcatheter surgery, and mini-invasive hybrid surgery—are discussed in the next session. The hybrid method is then comprehensively discussed with giving both success and complication rates compared to the other two approaches, as well as its clinical advantages and limitations. We also illustrate procedural aspects based on our own clinical experience. The mini-invasiveness, faster procedure, high success rate, and low incidence of post-operative complications in the hybrid approach provides good prospects for its wider use in VSD correction.

## 2 Ventricular septal defects

A ventricular septal defect (VSD) is an opening in the interventricular septum that permits abnormal blood flow between the left and right ventricles. This abnormal “shunting” increases pulmonary blood flow, and without treatment it generally leads to congestive heart failure. It was found that VSDs often accompany other CHDs (76).

According to most studies, perimembranous VSDs constitute the largest proportion of VSDs, often reported between 60% and 80% (77–79). Many perimembranous VSDs are hemodynamically insignificant and may not require intervention. Muscular VSDs are usually reported in 10%–15% of cases (80). A recent study performed in Malaysia also reported a greater proportion of perimembranous (55%) vs. muscular (30%) VSDs, with virtually similar occurrences of small and moderate sizes (42% and 46%, respectively) (81). On the other hand, a study involving 25,000 newborns in Denmark revealed a totally opposite distribution between the main types of VSD, with ∼93% having muscular VSDs and only 7% having perimembranous VSDs (82). These prominent discrepancies between reports may be due to several factors: the patients included in a study, regional (ethnic) variation in the prevalence of certain types of VSDs, or more accurate determination of smaller defects due to advanced diagnostic tools. Also, despite the predominance of perimembranous VSDs among diagnosed cases, muscular VSDs may account for a disproportionately higher part of interventional procedures due to their multiplicity or complexity.

Congenital VSDs constitute the vast majority of postnatally diagnosed VSDs, and acquired VSDs in newborns, children, adolescents, or adults are reported as relatively rare events (83). However, under certain conditions, like trauma, ischemic episode, or infectious myocarditis, acquired cardiac defects can occur in childhood and adulthood (84–86). Often, the acquired defect is a VSD in the form of a ventricular septal rupture, and in some cases, they can be repaired by the hybrid approach (87, 88).

Table 1 summarizes most often occurred VSD and their clinical considerations including associated risks.

**Table 1:** VSD types: prevalence, spontaneous closure, and clinical considerations.

VSD type	Prevalence	Spontaneous closure	Surgical indication	Associated risks
Perimembranous	70%–80%	Moderate	Common	Aortic regurgitation, heart block
Muscular	5%–20%	High	Rare (unless multiple)	Residual defects, arrhythmias
Inlet	5%–8%	Low	Common	AV valve involvement
Outlet	5%–7%	Low	Common	Aortic valve prolapse, regurgitation

Although many VSDs have a favorable prognosis, the clinical course is strongly influenced by defect size, subtype, and hemodynamic burden. The size of the ventricular septal defect may vary significantly. It is generally accepted that defects below 4 mm are small, between 4 and 6 mm are moderate, and above 6 mm are large (14, 89); however, a standardized size-based classification does not currently exist (73, 78, 90). Indeed, the nominal size of the VSD, as measured via an echocardiogram, provides little information about its actual functional role until the size of the heart is known. This is why another classification may be used, where the small–moderate–large scale for the VSD size is determined according to the ratio between the VSD size and aortic root diameter (91). According to this approach, an alternative classification for small, moderate, and large VSDs can be proposed: VSDs that are ≤1/3, >1/3 to ≤2/3, and >2/3 of the diameter of the aorta can be classified as small, moderate, and large, respectively (92).

Small perimembranous and muscular defects may close spontaneously either during the prenatal period or the first year of life, with muscular VSDs having a much higher closure rate than perimembranous VSDs (78, 82, 93). In younger patients aged <1–2 years, the rate of spontaneous closure of perimembranous or muscular VSDs varies widely, ranging from 30% (93, 94) to 55% (81) to 83.5% (82) within one or two years of observation. In longer observation periods, spontaneous closure rates may be as high as 97% for a 7-year follow-up period (89). A substantial proportion of prenatally discovered VSDs were found to close successfully in the uterus (95). A much higher rate of spontaneous closure after birth was reported for muscular VSDs compared to perimembranous VSDs (82, 89, 93); the difference between muscular and perimembranous VSDs is especially remarkable for sizes ≥4 mm (96). Of note, the higher likelihood for isolated muscular VSDs to close spontaneously correlates, to some extent, with a reportedly much lower frequency of chromosomal aberrations in these defects (0.4%) compared to isolated perimembranous VSDs (4.8%) (93). It is also not surprising that a smaller size appears to be one of the factors contributing to spontaneous closure (82) and that a larger VSD is a risk factor for post-operative complications and longer in-hospital stay (77).

In contrast, moderate-sized VSDs often necessitate surgical or catheter-based intervention, and large and very large VSDs always require urgent surgery (97). Moreover, the severity of VSD-related heart malfunctions generally depends not only on the nominal size of the VSD but also on the age of the patient (i.e., the size of the patient's heart). One study reported successful VSD closure surgeries in infants aged between 4.2 and 6.5 months, including some VSDs with very large sizes of ∼12–14 mm, according to the published data (98). In another study, two infant patients aged 3.5 and 3.9 months had even larger muscular VSDs, 14 and 16 mm, respectively (99). Note that these VSD sizes are comparable to those reported in adult patients (84, 100). In addition, large perimembranous or outlet defects are associated with progressive cardiac dysfunction, increased risk of endocarditis, and early development of pulmonary hypertension if left untreated (90, 101).

## 3 Conventional, transcatheter, and hybrid approaches for correction of ventricular septal defects

Management decision for VSD is guided by comprehensive risk stratification, incorporating defect size, location, shunt magnitude, and clinical symptoms (102, 103). Defects resulting in significant left-to-right shunting, chamber dilation, or pulmonary overcirculation require closure. Surgical or transcatheter closure may be contraindicated or technically infeasible in certain complex anatomies, such as multiple muscular VSDs or a single ventricle (104). Moreover, in younger patients, the surgical treatment of any type is accompanied by an increased rate of intraoperative cardiac arrest (105). It underscores the importance of individualized, anatomy-specific, and hemodynamics-related treatment planning. In this subsection, we discuss the surgical modalities that are used for VSD management (106–108): (a) conventional surgical repair utilizing cardiopulmonary bypass, (b) endovascular (percutaneous) closure techniques, and (c) hybrid VSD repair without the use of a heart-lung machine. These modalities differ in terms of their principles and implementation, and we briefly cover their historical evolution.

### 3.1 Conventional surgical repair of VSD utilizing cardiopulmonary bypass

Open-heart surgery using cardiopulmonary bypass (CPB) is considered the gold standard for VSD repair for a long time (109–112). This method is effective; however, it carries potential risks associated with the use of CPB and cosmetic-related issues. Technical advancements in the last decades have significantly improved patient compatibility with CPB. Nevertheless, the risk of systemic inflammatory response—manifested through edema, encephalopathy, thrombosis, or air embolism—still persists (113). Post-operative infections are another critical complication after CPB in pediatric patients, with some reports showing that nearly half of patients may develop secondary infection (114). Next, research indicates that the CPB procedure may impact neurodevelopment, especially motor skills, in infants—most likely due to the systemic stress induced by deeper and/or longer anesthesia (115, 116). The incidence of neurological complications in pediatric patients after CPB may be as high as 25% (117). Also, the presence of genetic anomalies is a risk factor for negative neurodevelopmental outcomes, as shown in infants aged <9 months at the time of CPB (115). In addition, post-operative pain due to full sternotomy and subsequent scar formation remains a significant concern (118, 119). It has therefore been concluded that CPB in children, particularly infants, is a challenging procedure affecting many physiological systems (117, 120).

Vertical right axillary and right anterior mini-thoracotomies (ministernotomy) are approaches mainly used as a standard approach to close atrial septal defects (ASDs), and can also be successfully and safely utilized for VSDs and atrioventricular septal defects (121–124). The implementation of these approaches, however, also requires the use of cardiopulmonary bypass, so their use in treating VSD brings increased risks for post-surgery complications. In addition, as discussed in the following section, these approaches almost always require a larger surgical incision compared to the hybrid approach, thus raising issues related to the cosmetic outcomes of the surgery.

These consequences, which affect patients’ quality of life, have prompted researchers to pursue innovations to improve surgical outcomes. As a result, efforts were initiated to develop interventional (percutaneous) methods to close ventricular septal defects.

### 3.2 Percutaneous (transcatheter) approach for VSD closure

The transcatheter approach for the closure of VSDs has been utilized since 1988, when Lock and colleagues reported the first percutaneous closure of a ventricular septal defect (VSD) in six patients, three of whom had post-infarction VSDs and three with congenital VSDs. At the time, this approach was considered an alternative treatment for selected patients (125). However, the endovascular method had its own limitations, including various types of arrhythmias, device embolization, and vascular complications. Nevertheless, with advancements in VSD closure devices, the first percutaneous closure of a VSD using the Amplatzer Occluder was performed in animal models in 1997, followed by successful application in patients with muscular VSDs since 1998 (126, 127).

The percutaneous transcatheter approach was shown to be effective in several clinical studies. For example, it was feasible in closing moderate and large perimembranous VSDs, with a success rate of 91.4% in 35 pediatric patients aged approximately 2 years and weighed below 10 kg (128). Similar results were found in children with weight deficiency, where the success rate was reported at 85.7% for the transcatheter closure of large or moderate perimembranous VSDs; the procedure did not result in mortality or any major complications during a median 20-month follow-up period (129). The transcatheter approach using a modified Amplatzer Duct Occluder II for the closure of small perimembranous VSDs was 100% effective and free from major complications, as shown in another study that involved 49 patients aged between 1.9 and 25 years (130). A single-center retrospective study that accumulated long-term data for 75 pediatric patients aged 46–96 months, who were treated via percutaneous closure using different types of devices (Amplatzer, Occlutech, Hyperion, etc.), showed a pooled 95.7% success rate for defect closure within 1 year after the treatment (131). Another single-center study retrospectively analyzed long-term outcomes for 149 patients, both children and adults, diagnosed with muscular or perimembranous VSDs and treated via transcatheter device closure; again, different brands and types of devices were used (100). The reported closure rate was 86.2% for a median 6-year follow-up period, with only one patient dying in the period due to the treatment procedure. Transcatheter closure was also shown to be effective in outlet-type VSDs, with 100% success closure and no incidence of complete atrioventricular block (cAVB) (132), and in post-myocardial infarction ventricular septal rupture (133). In general, according to systematic reviews on the effectiveness of transcatheter VSD closure, perimembranous VSDs can be successfully closed percutaneously in selected patients, with limited reports of major negative outcomes like regurgitation or heart block for at least one month post-treatment (134–136). Technical and procedural advancements allow for the successful implementation of the transcatheter approach in low-weight infants, mostly with perimembranous VSDs (137). According to a large meta-analysis collecting data from three randomized controlled studies and 24 observational studies (6,421 patients in total), comparison between open-heart surgery, transcatheter closure, and mini-invasive repair of perimembranous VSDs indicates the superiority of the transcatheter closure regarding the total duration of procedure, major complications, ICU stay, and in-hospital stay (135).

It is important to note that the entire procedure is performed under continuous fluoroscopic guidance, and the long-term effects of radiation exposure on various organs of the body remain insufficiently studied (138, 139). There are still numerous criteria that significantly limit the selection of suitable patients for this method (while mostly those who have ASD) (78, 104, 140). Additionally, complications such as injury to and regurgitation of the aortic and tricuspid valves have been reported (141–143), along with a high incidence of atrioventricular block or arrhythmias (136, 144–147). Further advancements in the approach, like single transradial arterial access, may partially overcome these challenges (148, 149). Also, using softer material for the device decreases the risk of conduction block issues, e.g., cAVB (103). However, the feasibility of routine use of percutaneous VSD closure in small patients (newborns and infants) is still debated; currently, it is advised to only use this method on selected pediatric patients (139, 150, 151). Therefore, despite the numerous reports on the utilization of the method for the surgical correction of VSDs, some limitations and challenges remain, e.g., in the correction of perimembranous VSDs (152).

### 3.3 Hybrid approach for VSD correction

An alternative approach is the hybrid procedure, known as transventricular or perventricular closure of ventricular septal defects. The term “hybrid procedure,” now commonly used in cardiology and cardiac surgery, refers to the combination of surgical and interventional techniques aimed at optimizing the treatment of congenital and acquired heart defects while minimizing their limitations. It is important to understand the rationale behind the term “hybrid”: this technique combines some features characteristic of open-heart surgery (e.g., making an incision in the thoracic region without the use of CPB) and catheter-based surgery to implant an occluder.

Initial attempts at hybrid VSD closure involved the direct use of CPB and employed the Rashkind double-umbrella occluder. However, the outcomes of these early procedures were suboptimal, with failure rates ranging from 20 to 40% and mortality rates between 14% and 25% (153–156). The first successful implantation of an occluder on a beating heart without CPB was performed in 1998 by Dr. Amin and his colleagues (157). In animal-based experimental studies, muscular VSDs were artificially created in pigs and dogs to advance the technique of VSD closure on a beating heart (157–160).

As technology evolved, self-centering implants became popular, allowing for more flexible devices to be used. Furthermore, the use of transesophageal or epicardial echocardiography significantly improved the accuracy of diagnosis and treatment. The first promising studies on hybrid VSD closure emerged after the first half of the 2000s (161, 162). Moreover, researchers sought alternative incision techniques for VSD closure. In 2003, Bacha and colleagues reported a case utilizing a subxiphoid incision, marking the first instance of a muscular VSD being closed without CPB via this approach (163). Later, perimembranous VSD closure without CPB was successfully applied in over 400 patients, aged between 5 months and 15 years, with an overall success rate of 96.3% and only ∼3% of patients experiencing minor adverse effects like tricuspid valve regurgitation or an incomplete right bundle branch block (164). Other studies reported successful closure rates between 90% and 100% in infants and children with low weight (165–168). Of note, the success closure rate may be below 100% immediately after the surgery but can eventually achieve this rate, e.g., 6 months later (166).

Based on available published data and our own gathered experience, the average mortality rate associated with the hybrid approach appears to be lower than that observed with conventional surgical correction using CPB or with the percutaneous transcatheter approach. The available reports often indicate no mortality within short- and medium-term periods. For example, in a small cohort of 30 pediatric patients (all with non-muscular VSDs, aged <3 years or weighed <15 kg), no peri- or post-mortality was observed during a 6-month period (165). The hybrid approach was applied to another small cohort of infants aged <1 year with a large apical muscular VSD of mean size 8.5 mm, and no fatal cases during 36-month follow-up were reported (169). Many other case reports, case-series reports, and large retrospective analyses also provide data indicating no mortality in the hybrid method for VSD closure in patients of different ages, from neonates to adults (167, 170–172). In patients with post-myocardial infarction VSDs, the early mortality was substantially higher in the open-heart surgery group with CPB; however, the 1-year mortality was similar in these groups (173). Our own data accumulated from 500 pediatric patients subjected either to hybrid or conventional open-heart surgery—250 in each group—indicated much lower in-hospital mortality for the hybrid approach (0.43% for all-age mixed data) vs. traditional open-heart surgery with CPB (1.5%) (174). However, there is still the need to accumulate long-term results for survival rates and, in particular, any delayed unfavorable effects, including neurological and developmental disorders.

Indeed, it is well known that left ventricular (LV) function, in most cases, is compromised immediately after VSD repair and in the short-term period (175, 176). Moreover, in middle- or long-term periods, the LV function may be restored incompletely. For instance, a clinical study involving infants younger or slightly older than 6 months (in total 104 patients) showed that the post-operative LV dysfunction was observed in 38% of the patients and that the recovery of LV contractility requires ∼9 months (177). Similar estimates were obtained in another study, where pediatric patients with pre-operative LV dilation, aged one year or younger, needed approximately 12 months to recover their LV function to the pre-surgery baseline (178). It has also been found that the elevated pre-operative internal dimension of the LV is a risk factor for either the post-operative LV dysfunction occurrence or the prolonged recovery in LV contractility (176). The negative post-surgery outcomes may relate not only to the LV ejection fraction (LVEF, used as a standardized functional measure of the pumping function of the ventricle) but also to its mechanical deformability measures, even in the case of normal LVEF values (179). On the other hand, these reports mostly concern the patients treated by conventional surgery with CPB, with, in general, much longer anesthesia and operation time. Yet, comparing the conventional open-heart surgery and transcatheter device closure within a single study revealed eventual recovery of LV function in both groups, despite a more pronounced decrease in LV function being detected in those subjected to open-heart surgery (180). Of note, a substantial decrease in LV function is of particular concern in small children, even when being treated for small defects (181).

Additionally, right ventricular (RV) dysfunction may occur after VSD repair (182–185), although many studies did not report its occurrence (186, 187) or observe some improvement in RV function in patients with VSD after post-myocardial infarction (188–190). Although long-term studies on the hybrid method are still lacking and some show single cases of serious adverse effects like cAVB (103, 191), short-term data indicate that serious post-operative functional complications are rare. Furthermore, when treatment is conducted in early childhood, patients appear to have normal LV function, i.e., comparable to that of healthy individuals (192).

Perventricular VSD device closure is mostly used in patients with muscular VSDs (165, 166). Also, it has been validated as a safe approach in complex muscular VSDs in infants (193). Moreover, the perventricular approach has been reported as a successful approach for the closure of perimembranous or doubly committed VSDs (111, 167, 194, 195). In infants and patients with low weight, a subxiphoid perventricular approach via small incision was found to be a successful treatment in ∼90% of patients (*n* = 17), including the closure of muscular and perimembranous VSDs (196). In another study with 21 individuals, including seven neonates, no post-operative mortality, morbidity, need for reoperation, and functional consequences were reported after the perventricular hybrid approach (197). A larger cohort of 59 patients, mostly with perimembranous VSDs, was treated via the perventricular approach, with an overall successful closure rate of 97% and only one major post-operative complication (167). Others report similar good outcomes for perimembranous, muscular, and subaortic VSDs in infants, with no observed late rhythm disturbances or other complications (111). Small infants with large defects and patients with insufficient venous access are the selected patients for the perventricular hybrid approach, including muscular VSDs (194, 198). In addition, a hybrid perventricular transcatheter approach may be successfully performed in patients with apical VSD developed after myocardial infarction, with much better in-hospital survival compared to traditional surgery (173).

In addition to generally better values for mortality and post-surgery complications, the hybrid approach also has advantages due to its shorter operation duration and overall cost-effectiveness. Many reports show that the procedural duration is nearly two times shorter in hybrid vs. traditional surgery (120, 170, 174, 195, 199). Mini-invasive surgical treatments in pediatric patients, particularly those with congenital heart defects, continue to develop, and further assessments regarding their safety, outcomes, and long-term efficacy (evaluated by care-related costs, everyday activity, etc.) will be conducted (200).

## 4 Procedural and technical aspects of hybrid approach in treating ventricular septal defects

**Figure 1:**
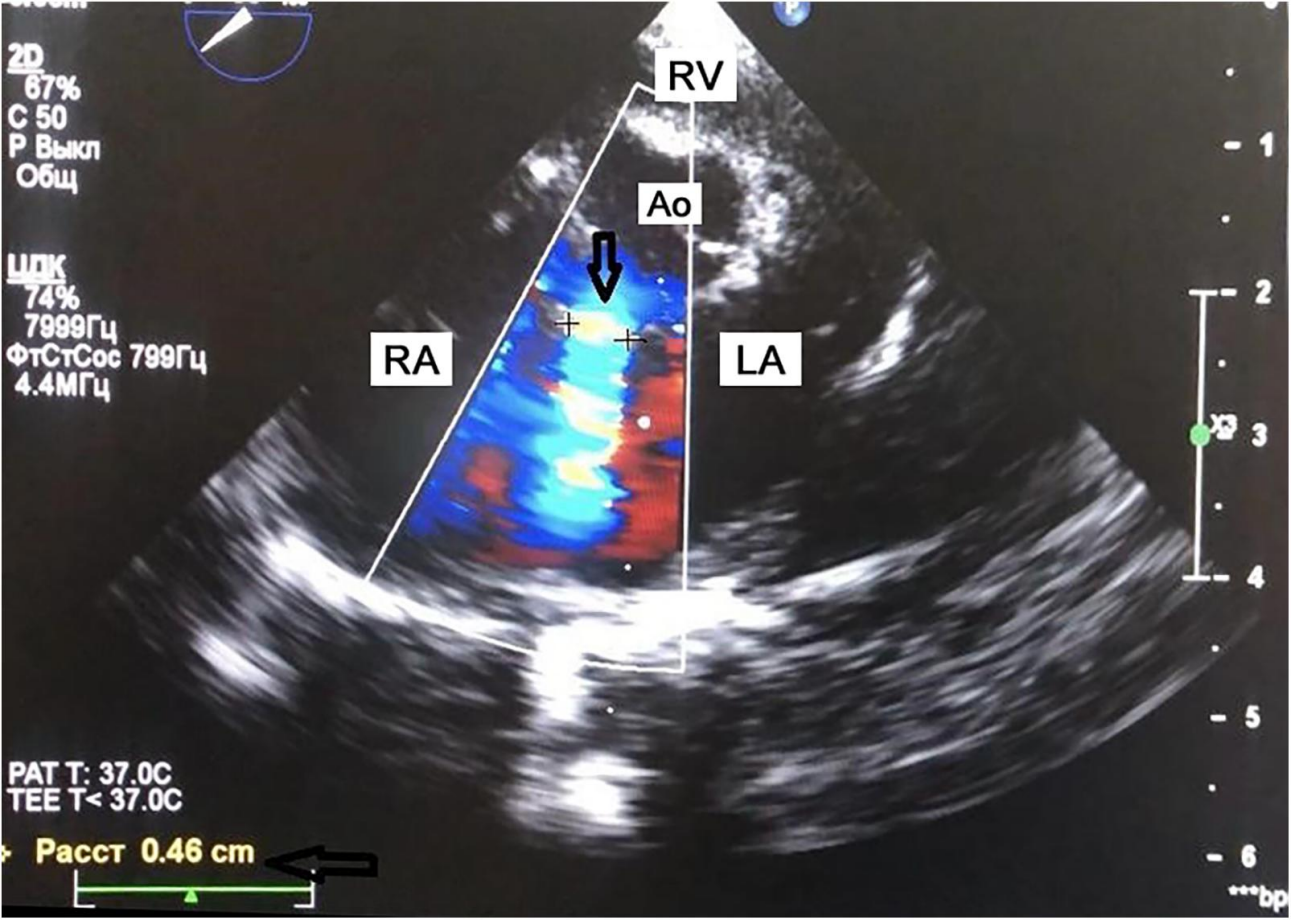
Short-axis view of ventricular septal defect via transesophageal echocardiography (TEE). The arrow points to the ventricular septal defect and shows its approximate size. In the lower left corner, the defect is visualized with a size of 0.46 cm. This is one of the positions used for the final selection of the type and size of the occluder. Traditionally, it is enough to use two positions: along the short axis (with an angle of 30–50 degrees) and along the long axis (with an angle of 120 degrees). This gives the maximum information about the defect in order to select the right occluder for it. RA, right atrium; Ao, aorta, RV, right ventricle; LA, left atrium.

**Figure 2:**
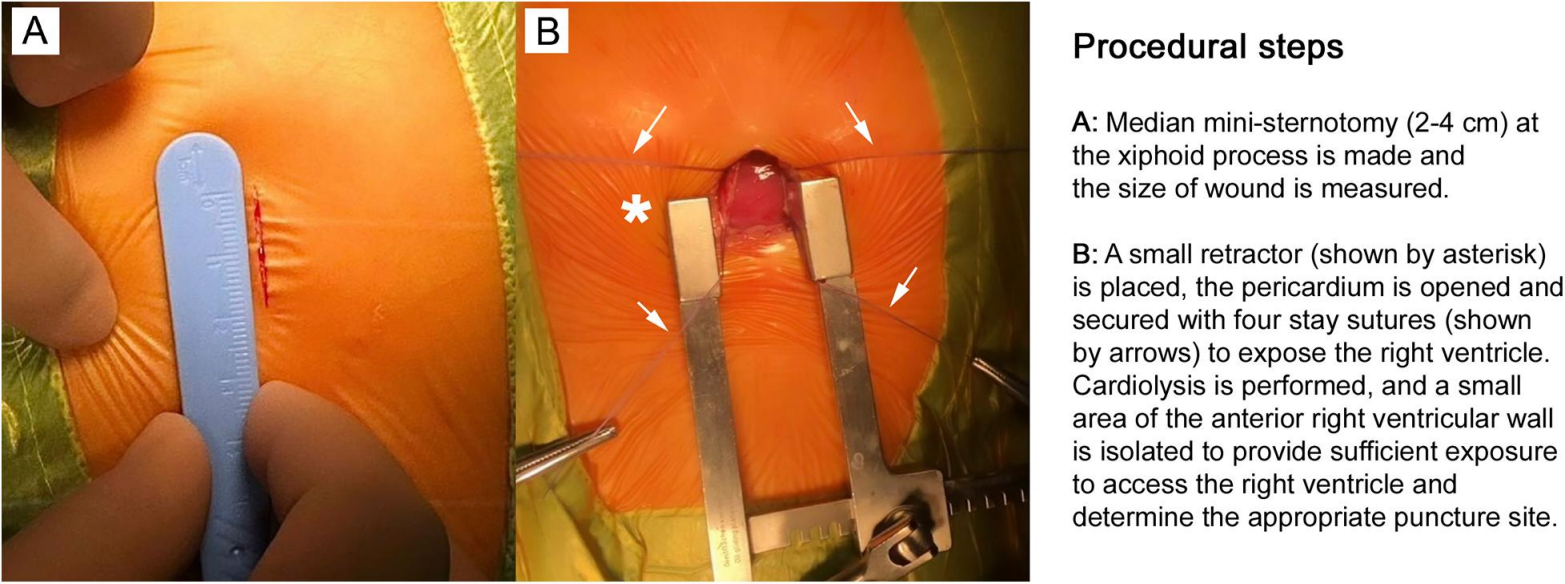
Initial procedural steps. **(A)** Typical surgical wound size in the hybrid method. **(B)** Access is obtained through a 2–4 cm median mini-sternotomy at the xiphoid process. A small retractor (shown by asterisk) is placed, and the pericardium is opened and secured with four stay sutures (shown by arrows) to expose the right ventricle (RV). Cardiolysis is performed, and a small area of the anterior right ventricular wall is isolated. This provides sufficient exposure to access the RV and determine the appropriate puncture site. The images are taken in our own surgery department, hybrid room, 11-month-old patient with perimembranous ventricular septal defect.

**Figure 3:**
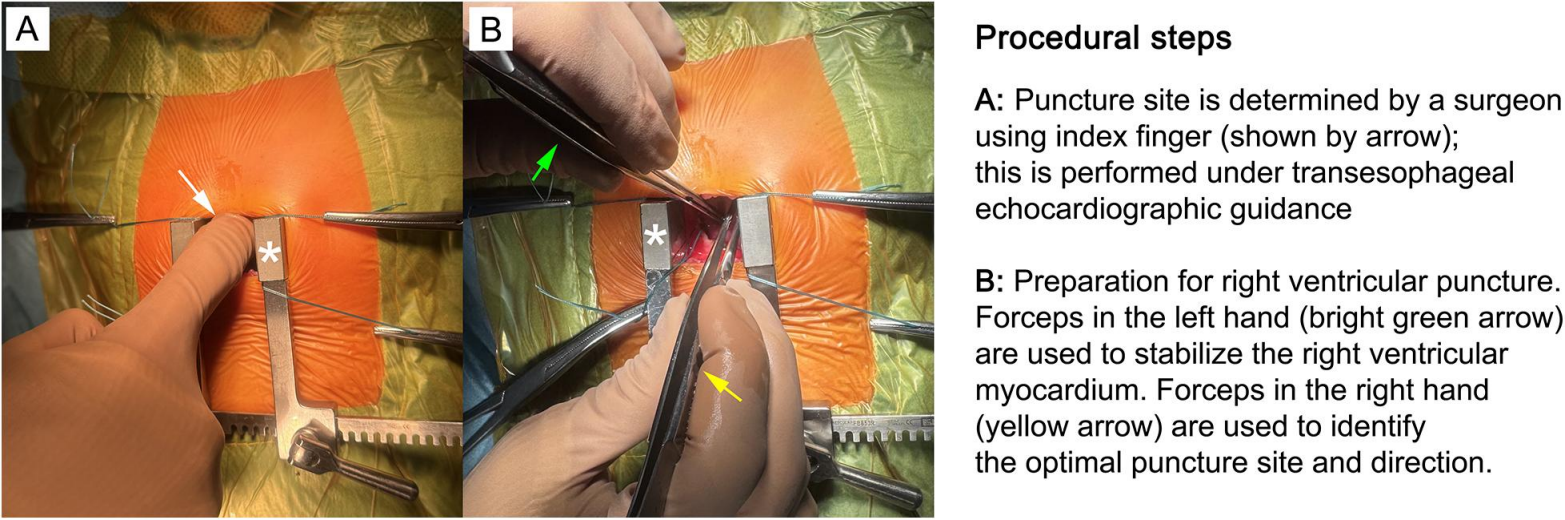
Methods for determining the correct location for right ventricular (RV) puncture. **(A)** Using the right index finger (shown by arrow), a surgeon identifies the approximate puncture site under transesophageal echocardiographic (TEE) guidance. **(B)** With the left hand, the surgeon uses forceps (bright green arrow) to stabilize the RV myocardium; forceps in the right hand (yellow arrow) are used to identify the optimal puncture site and direction. The surgeon can apply some pressure with tweezers at the point of the intended puncture of the RV. The trajectory of the guide and the angle at which the guide will approach the VSD are visible on the echocardiogram monitor. All these manipulations allow choosing the optimal puncture site and, thus, achieving the best result, avoiding repeated puncture or mechanical injury to the wall of the interventricular septum during the passage of the guide into the defect. The retractor is shown by asterisk on both panels. The images are taken in our own surgery department, hybrid room, 11-month old patient with perimembranous ventricular septal defect.

**Figure 4:**
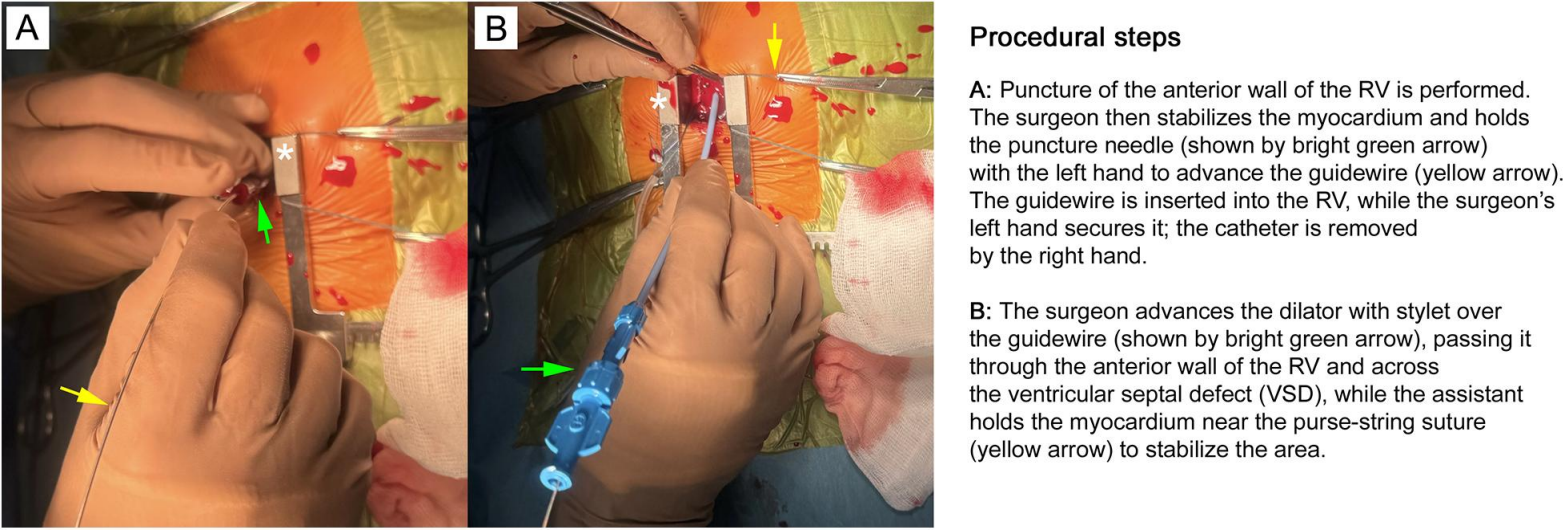
Right ventricular (RV) puncture, installation of a guidewire and dilator. **(A)** Puncture of the anterior wall of the RV is performed. The surgeon then stabilizes the myocardium and holds the puncture needle (shown by bright green arrow) with the left hand to advance the guidewire (yellow arrow). The guidewire is inserted into the RV, while the surgeon's left hand secures it, and the catheter is removed by the right hand. **(B)** The surgeon advances the dilator with stylet over the guidewire (shown by bright green arrow), passing it through the anterior wall of the RV and across the ventricular septal defect (VSD), while the assistant holds the myocardium near the purse-string suture (yellow arrow) to stabilize the area. As soon as the dilator advances over the guidewire into the left ventricular (LV) cavity, the surgeon releases the myocardium and removes the stylet along with the guidewire, while the assistant maintains the same position. This stage is considered extremely important. The surgeon's precise movements allow him to pass a rigid stylet into the defect lumen at the very first attempt without damaging the wall of the interventricular septum, where the cardiac conduction system is located near the defect crest. This helps avoid heart rhythm disturbances, which are the most serious complications with this surgical technique. The second important point is the possibility of damaging the body of the aortic valve leaflet itself with a straight rigid stylet, which can lead to its insufficiency. Therefore, each movement of the surgeon must be strictly controlled by the transesophageal echocardiographic (TEE) guidance. The retractor is shown by asterisk on both panels. The images are taken in our own surgery department, hybrid room, 11-month old patient with perimembranous VSD.

**Figure 5:**
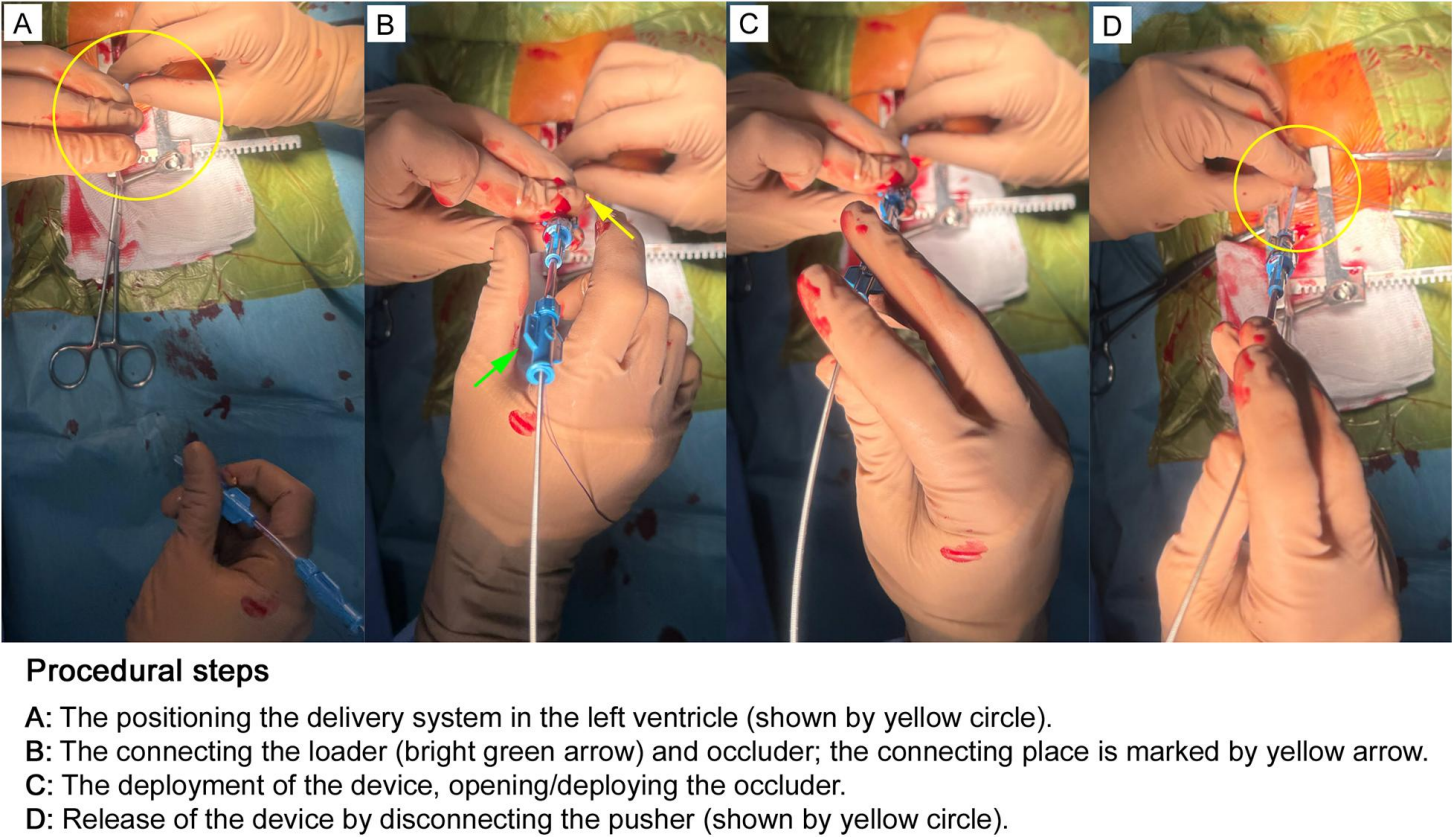
After placing the delivery system in the left ventricle, the loader and occluder are connected [**(A,B)**, shown by yellow circle and yellow/green arrows]. The surgeon positions the right hand to control the system: the fourth and fifth fingers stabilize the delivery system, the third finger marks hand position, and the first and second fingers advance or retract the loader and occluder. The operator grasps the myocardium near the system while the assistant relaxes it, allowing precise heart positioning during occluder deployment and preventing displacement when angled to the septum. The occluder is opened **(C)**, and then the pusher is disconnected [**(D)**, the process of disconnection is shown by yellow circle]. After careful analysis of the occluder location using the transesophageal echocardiographic (TEE) guidance and the hemodynamic and ECG parameters on the anesthesia monitor, the surgeon performs the final stage of implantation, i.e., disconnecting the delivery system from the occluder. The images are taken in our own surgery department, hybrid room, 11-month old patient with perimembranous ventricular septal defect.

**Figure 6:**
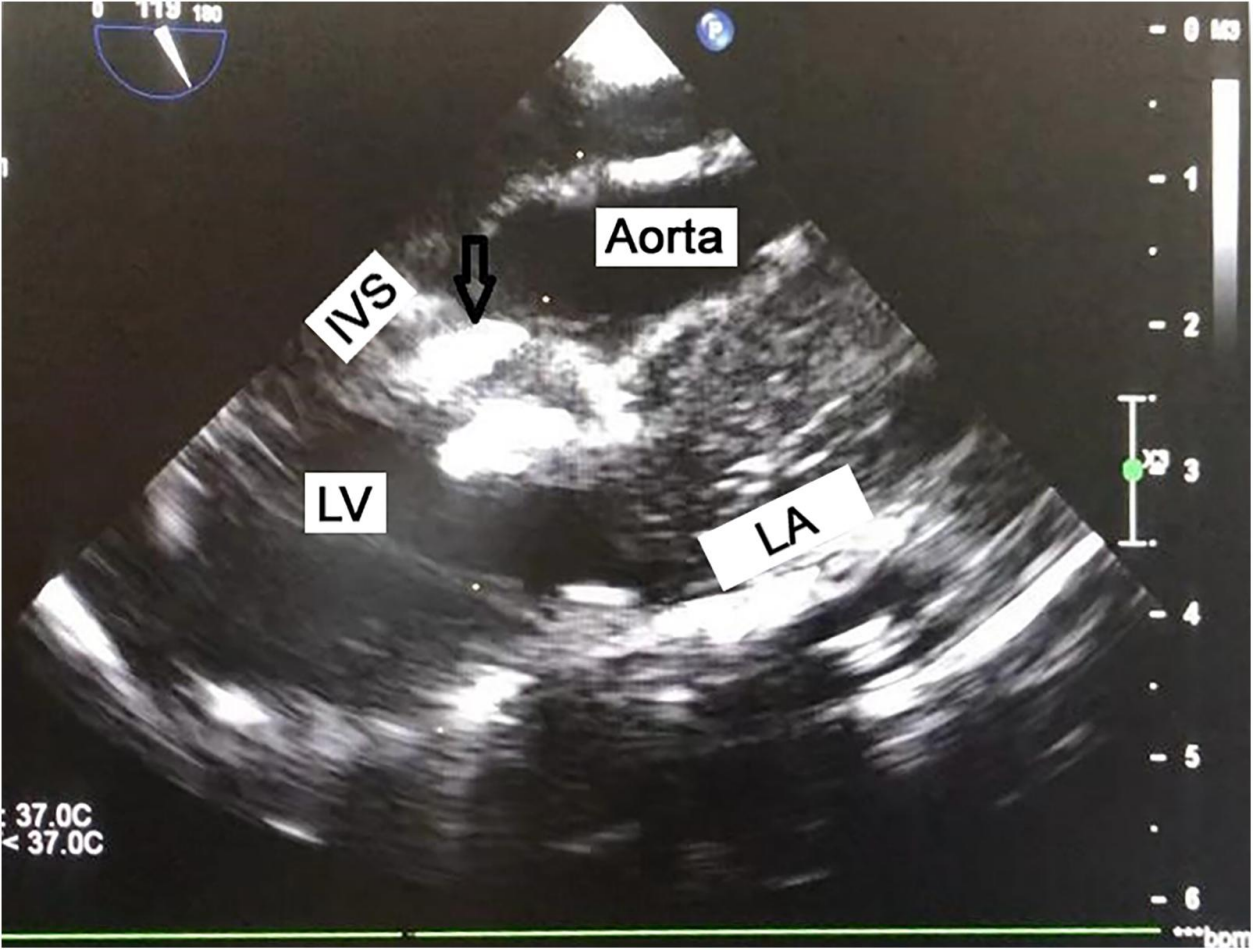
The occluder in the installed position (B-long-axis view). The black arrow shows the opening of the left and right occluder discs. The surgeon has the ability to relocate and reopen the discs if there is any doubt about the success or correctness of the device installation. There is always the option of replacing the occluder with one of a different type and size. IVS, Interventricular septum; LV, left ventricle; LA, left atrium.

**Figure 7:**
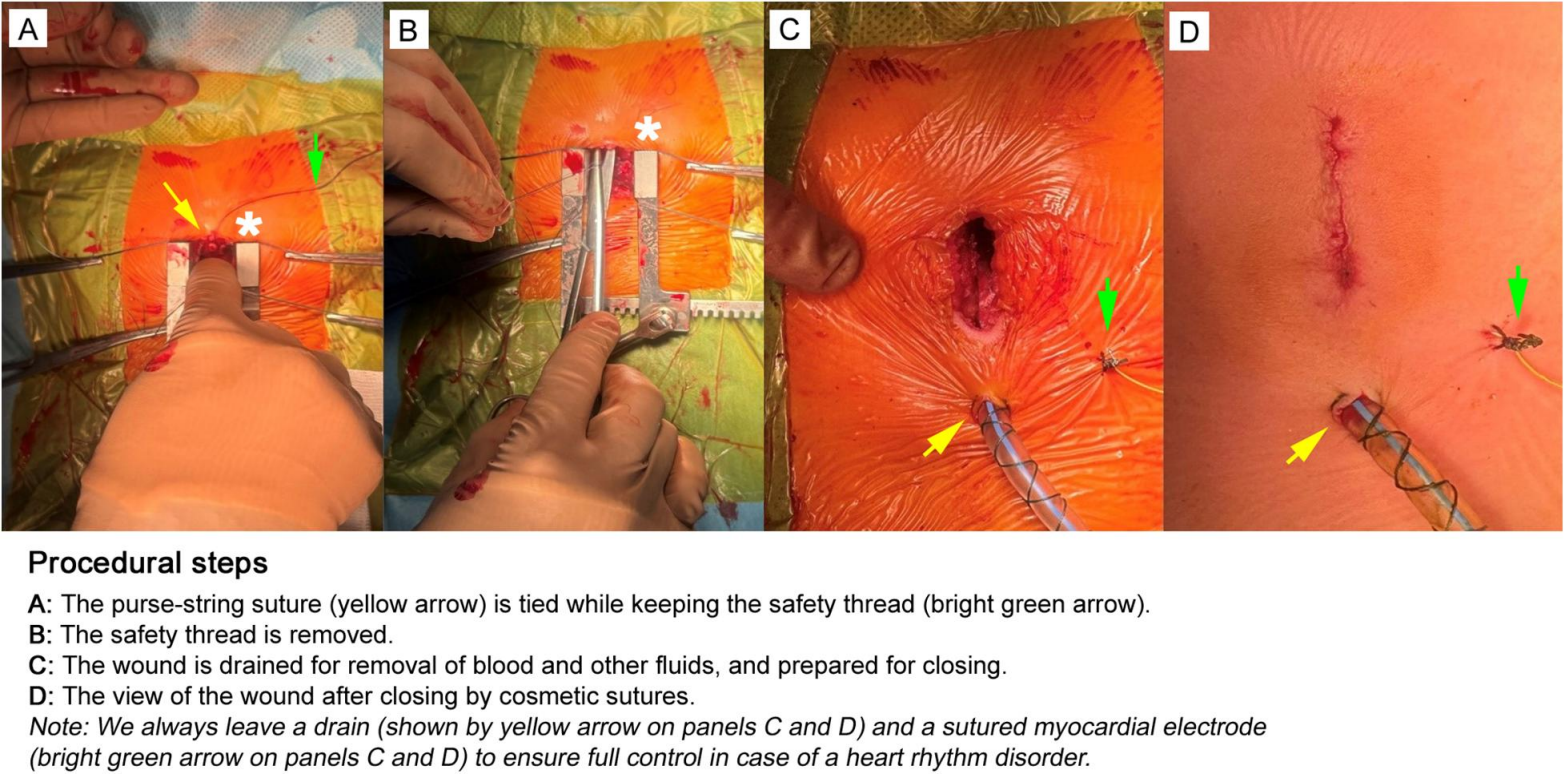
The final procedural steps for closing the surgical wound. **(A)** The purse-string suture (yellow arrow) is tied while the surgeon uses the index finger to gently stabilize and control the tissue, ensuring accurate placement and preventing the suture from tearing through the myocardium; the safety thread is shown by bright green arrow. **(B)** The safety thread is removed. The surgical field is carefully inspected to confirm homeostasis, proper positioning of all leads, and the absence of residual debris. **(C)** The wound is drained for removal of blood and other remaining fluids, and prepared for closing. **(D)** The view of the wound after closing by cosmetic sutures. We always leave a drain (shown by yellow arrow on panels C and D) and a sutured myocardial electrode (bright green arrow on panels C and D) to ensure full control in case of a heart rhythm disorder. The retractor is shown by asterisk on panels A and B. The images are taken in our own surgery department, hybrid room, 11-month old patient with perimembranous ventricular septal defect.

The hybrid approach to ventricular septal defect closure integrates surgical exposure with interventional devices on a beating heart, eliminating the need for cardiopulmonary bypass. This procedure is conducted under general anesthesia with continuous transesophageal echocardiographic (TEE) guidance. The primary procedural steps are outlined below and illustrated in Figures 1–7.

### 4.1 Surgical steps of the hybrid ventricular septal defect closure technique

#### 4.1.1 Step 1: preoperative assessment

Preoperative TEE is essential for accurate assessment of VSD size, location, and morphology, with special attention to any associated valvular abnormalities (Figure 1). Based on this evaluation, an appropriate occluder is selected—typically symmetric, asymmetric, eccentric, or muscular types—tailored to the defect anatomy.

#### 4.1.2 Step 2: surgical access

A 2–4 cm inferior median sternotomy is performed, followed by a pericardiotomy to expose the anterior free wall of the right ventricle (RV). For subarterial VSDs, an anterior parasternal incision may be used as an alternative approach (Figure 2).

#### 4.1.3 Step 3: right ventricular puncture

Under real-time TEE monitoring, the optimal puncture site on the RV free wall is identified (Figures 3A,B). A purse-string suture is placed around the selected site, and a trocar or 6F needle is utilized to puncture the RV wall.

#### 4.1.4 Step 4: guidewire and sheath insertion

A 0.035-inch guidewire is introduced into the RV and passed across the VSD into the left ventricle (LV). The trocar is then removed, and a delivery sheath is advanced over the guidewire into the LV cavity (Figure 4).

#### 4.1.5 Step 5: device deployment

With the heart beating and under continuous TEE guidance, the occluder is delivered through the loading sheath (Figure 5). The left ventricular disc is deployed first against the interventricular septum, followed by deployment of the right ventricular disc, achieving secure closure of the VSD.

#### 4.1.6 Step 6: post-deployment assessment

After successful device release, intraoperative TEE is used to evaluate residual shunts, device position, and potential complications such as aortic or tricuspid regurgitation (Figure 6). Special attention is given to detecting conduction abnormalities such as an atrioventricular block. If significant residual shunting (>2 mm), new-onset valve dysfunction, or conduction disturbances are observed, conversion to conventional on-pump surgery may be required. The delivery system is detached only after favorable transesophageal echocardiography (TEE) findings: no residual shunt across the ventricular septum, preserved aortic and tricuspid valve function, and absence of rhythm or conduction disturbances. A safety suture remains in place and is exteriorized through the purse-string suture. After securing the purse-string, TEE is repeated to reassess the heart. The safety suture is then shortened, the knot is tied, and the sternotomy is closed (Figures 7A,B). Hemostasis is verified, and protamine sulfate is administered if needed. In primary procedures, stainless steel wires are not used for sternal closure, whereas in reoperations, they are applied. If indicated, epicardial pacing wires are placed. Standard pericardial drainage is maintained for 24 h (Figures 7C,D).

This algorithm provides the optimal level of automation and control of unnecessary movements, which allows for minimizing trauma and reducing the total duration of the surgical procedure. This sequence of actions is based on our eight years of experience and vision of the operation, which, in turn, has demonstrated high efficiency.

### 4.2 Device delivery approaches

**Figure 8:**
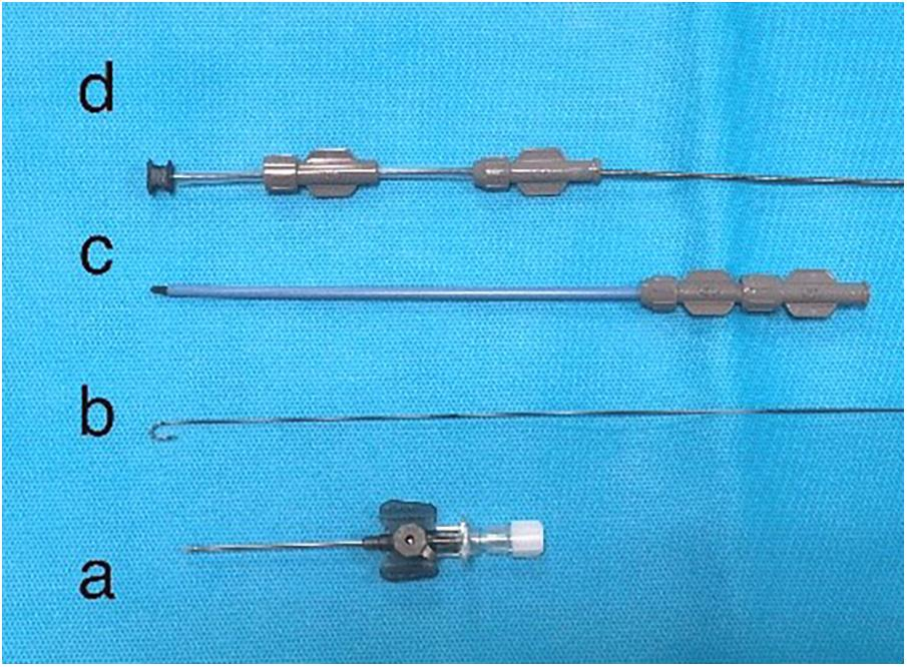
Delivery system for VSD occluder consists of the following: **(a)** trocar (puncture needle); **(b)** guidewire; **(c)** delivery sheath and dilator; **(d)** loading sheath with a muscular occluder.

An occluding device can be delivered either directly to the ventricle via a small incision in the heart's chamber or via a large vessel like the femoral vein using a catheter-based delivery system. The system consists of the following: (i) the VSD occluder, designed to connect to the delivery cable via a screw mechanism; ((ii) the guidewire, used to advance the devices into the desired position (Figure 8B); (iii) the dilator, which facilitates penetration through tissue and the vessel wall; (iv) the hemostatic valve, which minimizes bleeding, and includes a side port with a flexible extension tube and a stopcock used for flushing the system (Figure 8A); (v) the loading sheath (loader), which is used to introduce the occluder, attached to the delivery cable, into the introducer (Figure 8D); and (vi) the delivery sheath with plastic torque control (Figure 8C). The delivery sheath is used to advance the occluder through the introducer and hold it in place while the introducer is retracted to deploy the occluder or retrieve it if its size, position, or deployment is deemed unsatisfactory. The plastic torque device, screwed onto the proximal end of the delivery cable, facilitates directional control and serves as a handle for detaching the delivery system from the occluder.

### 4.3 The types of devices used to close VSD

**Figure 9:**
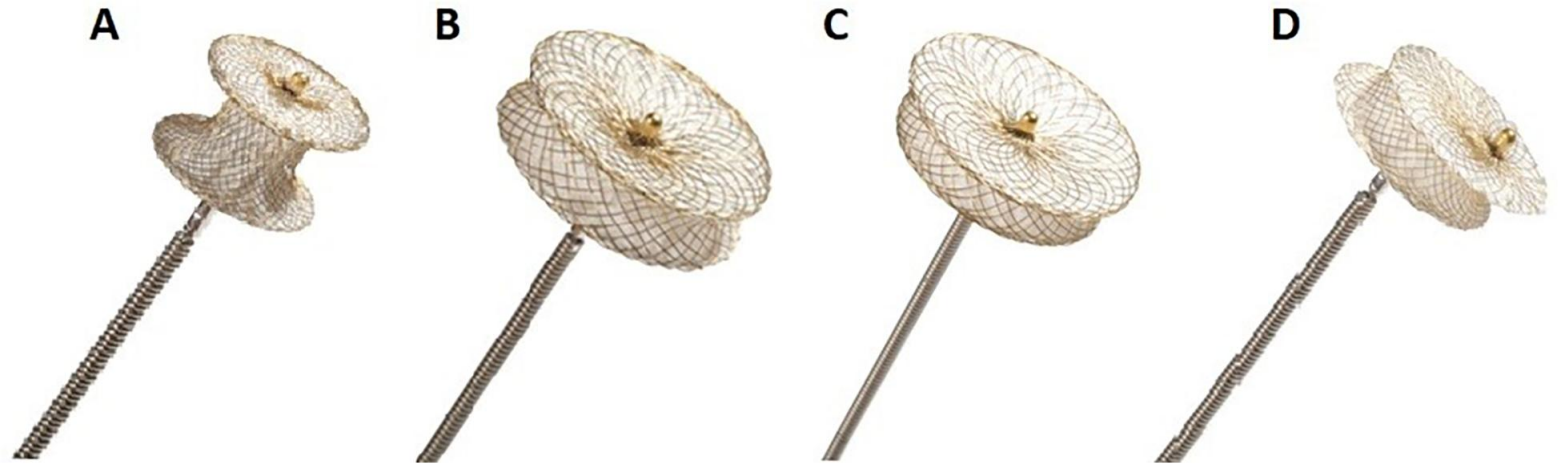
Types of occluders used for VSD closure (cera occluders, lifeTech scientific Co). **(A)** Muscular occluder. **(B)** Symmetric occluder. **(C)** Asymmetric occluder. **(D)** Eccentric occluder. Today, there are a large number of occluder manufacturers, mostly from China. Different manufacturers introduce their own details into the generally accepted design. For example, occluders without a second metal head have less turbulence in this area, thereby reducing the risk of hemolysis and thrombus formation. Currently, clinical trials of absorbable occluders are actively being conducted, which, according to manufacturers, after the formation of the interventricular septum wall from the body's own cells, lose their own structural components and, after 2 years, do not remain in the human body at all. The studies in this context are ongoing. Reproduced from “Types of occluders for closure VSD (Cera Occluders, LifeTech Scientific Co).22 A, Muscular occluder; (B) symmetric occluder; (C) asymmetric occluder; (D) eccentric occluder" by Akkerbez Adilbekova, Shukhrat Marassulov, Abay Baigenzhin, Saken Kozhakhmetov, Bakhytzhan Nurkeyev, Amangeldy Kerimkulov, Saniya Murzabayeva, Rinat Maiorov and Arailym Kenzhebayeva, licensed under CC BY-NC-ND.

The occluders used for VSD closure may have different shapes and sizes (Figure 9) and the are used for different types of VSD and indications as listed in Table 2. The most common types of occluders used in VSD closure are concentric and eccentric. Generally, the former is used more frequently compared to the latter. For example, the concentric type was used in >70% of devices while the eccentric type was used in 25.6% cases of <1 year old infants with muscular and perimembranous VSDs (201). Special types of occluders can also be used, but their prevalence has been reported as low as 5% of the total number of occluders (201). Typically, the size of the VSD occluder is 2–3 mm larger than the diameter of the VSD (the waist of the occluder corresponds to the VSD diameter) (202). Transcatheter implants have unique material properties, including shape memory. Therefore, the devices for the closure of VSDs (and other types of defects) are pre-shaped according to the individual anatomical features. Importantly, these devices can be compacted, delivered through catheters, and deployed intracardially, where they resume their intended shape.

**Table 2:** The list of VSD occluders and corresponding types of VSD and indications for which any type of occlude is generally used.

Types of occluders	Indications/when used
Muscular (see Figure 9A)	For muscular VSDs located in the trabecular (muscular) septum and fully surrounded by myocardium.
Symmetric (see Figure 9B)	For perimembranous VSDs with adequate rims, especially a good aortic rim, when both sides of the defect allow symmetric disc placement.
Asymmetric (see Figure 9C)	For perimembranous VSDs close to the aortic valve. The asymmetric design prevents pressure on the aortic valve.
Eccentric (see Figure 9D)	For VSDs with a deficient rim near critical structures, mainly the aortic valve. Eccentric design shifts the disc away from the valve.

According to many available reports, the most frequently used VSD occluder in the periventricular approach is the Amplatzer Muscular VSD Occluder (194). This device is a self-expandable double-sided disc made of nitrinol wire mesh and polyester, providing tight deployment at the site of the defect and space for surrounding tissue to grow inside the device. Older versions of Amplatzer closure devices for perimembranous VSD are no longer used in some countries, such as the U.S., due to the high risk of a complete heart block; however, they are widely used in other countries (203). Currently, widely used essential analogs of Amplatzer Occluders include Konar MFO (79, 204–206) and MemoPart™ (111, 199, 207). Moreover, the off-label use of different variants of Amplatzer or Amplatzer-like devices, specifically in the closure of VSDs, was shown to be feasible in selected patients. The examples include closure of perimembranous VSDs (130, 208–210) or subarterial VSD (211) via Amplatzer Duct Occluders (ADO II and ADO I) and closure of perimembranous VSD via the Amplatzer Vascular Plug II (212, 213). Notably, the off-label use of the occluder results in a fairly good success rate according to these reports. In some reports, it has been shown that the use of duct occluders instead of the original VSD occluder is safer as it avoids cAVB (208). In addition, compared to the conceptually different and widely used type of occluding device, Nit-Occlud Lê VSD Coil, the Amplatzer and Amplatzer-like Duct Occluders show somewhat better occlusion success rate (214, 215). For example, a comparative study showed that six months after the occlusion procedure, the complete occlusion was remarkably higher, although non-significant, in the group with duct occluders compared to the coil group: 91.3% vs. 84.1%, respectively (214).

A novel transcatheter device occluder, LifeTech™ Multifunctional Occluder (also known as Konar-MFO), was introduced several years ago as a promising closure device for muscular VSDs (171). It has recently been evaluated for its safety in terms of avoiding intra- or post-operative atrioventricular blockage (206). The meta-analysis thoroughly compared 19 studies that used this device, and it was found that the overall success rate was >94%, with cAVB reported in only 2.3% of followed-up patients. In a recent clinical study, percutaneous closure of muscular or perimembranous VSDs via Konar-MFO was implemented in 151 patients, with a total successful closure rate of 98.7%; however, in 3.3% of the patients there rhythm disturbances were observed (79). In 57 patients subjected to percutaneous VSD closure via Konar-MFO, a slightly lower success rate, 93%, was reported in another clinical study, but no complications related to rhythm disturbance were observed within a 6-month follow-up (205). Only one case of complete heart block was reported in a clinical study on 98 patients (mostly pediatric) with perimembranous and muscular VSDs subjected to Konar MFO device deployment, whereas the overall success of the procedure was 98% (203). A smaller set of five patients with perimembranous VSDs, aged <12 months, was surgically repaired via Konar-MFO, and no electric block of the heart was observed during a 15-month follow-up (2). Importantly, the feasibility of Konar-MFO has recently been demonstrated in low-weight infants (below 10 kg), with procedural advancement where no arterial access and transesophageal echocardiographic guidance were necessary, and only the venous route was used for catheterization (137). However, the lack of long-term follow-up observations still exists, and there are some reports about adverse events after using Konar-MFO as late as 20 months post-surgery; on the other hand, this case involved the closure of perimembranous VSD using a transcatheter device (216).

The effectiveness of Konar-MFO was directly compared to the commonly used Amplatzer Duct Occluder II device: long-term outcomes for 33 vs. 44 patients were analyzed, respectively (204). There was no reported permanently persisting heart conduction block or death in both groups, and the freedom from post-operative complications was high and comparable between these devices. On the other hand, some studies indicate that the Konar MFO device is more frequently used in larger VSDs. According to available comparative studies for these two devices, the difference in average or median defect sizes may be as high as 2.5 mm (204, 217). There are several ongoing studies that continue accumulating data about the safety of Konar-MFO and long-term outcomes for post-treatment complications (103).

Another novel device, the Cocoon Membranous VSD Occluder, has recently been introduced into clinical practice, and it is currently utilized for the correction of perimembranous VSDs. This is a symmetric fabric-reinforced double-disc device made of nitrinol designed for compact closure of a defect (218). Preliminary reports were concerning its feasibility in pediatric and/or adult patients with VSD (219), including post-operative defects (220). In recent studies, the effectiveness and the prevalence of the Cocoon Duct Occluder have been compared to other types of occluders. For example, it was revealed that the Cocoon Duct Occluder is the most prevalently used device in India for the closure of perimembranous VSDs (210). However, early reports show that the implanting of this type of device may be accompanied by adverse effects, including leakage and an incomplete conduction block (219). It is therefore needed to gather additional data on the safety and effectiveness of Coccon devices in further studies.

Finally, some most recent studies utilize a principally novel type of occluders made of bioabsorbable material (221, 222). These studies are at the initial stage for evaluation of late consequences and generally provide the assessment for up to 1-year follow-up period and are performed in relatively small cohorts of patients. Unlike nitinol-based occluders which may result in some late complication like atrioventricular block, the biodegradable occluders are considered safer in this context (223). The perspectives for using this novel type of VSD (and other types of defects) occluders will be refined during accumulation of enough amount of clinical data needed for careful assessment of late complications and complete occlusion of defects.

In our center, we use CeraTM Occluder (LifeTech Scientific Co., China). It is a self-expandable, double-disc device crafted from nitinol wire mesh. The two discs are interconnected with a short cylindrical waist tailored to match the size of the VSD. Both the discs and the waist feature polytetrafluoroethylene (PTFE) membranes securely sewn to the device using nylon threads. It has been shown in early studies that CeraTM/CeraFlexTM septal occluders are safe and are a good alternative to Amplatzer devices in treating ASD and PDA via a transcatheter approach (224–227). Nevertheless, similar to Cocoon occluders, further studies are needed for further verification of the long-term safety of this type of device.

To conclude, various types of VSD occluders are used by different teams/centers; there is also regional variation in using various VSD occluders. The effectiveness and safety of the above-mentioned VSD occluders were directly compared only in a few studies. Moreover, novel types of VSD occluders like biodegradable ones require accumulating enough amount of clinical application, especially in low-weight children, to perform type-to-type comparison with traditional nitinol-based occluders.

## 5 Advantages and limitations of hybrid approach for VSD closure

One of the advantages of the hybrid approach is that it is performed on a beating heart, i.e., without the need for cardiopulmonary bypass (227). Another important advantage of hybrid closure is its feasibility in very small patients, often weeks or even days old (78). Indeed, the hybrid approach has demonstrated good applicability and feasibility in neonates and infants aged <1 year, according to several clinical studies (196, 197). The perventricular hybrid approach is especially effective in small infants with large defects or in patients with insufficient access through the vasculature (194, 228). Third, the overall duration of the hybrid treatment surgery is substantially shorter compared to conventional surgery. Thus, some studies have reported that hybrid approaches have mean procedural and anesthesia durations that are two to three times shorter than conventional approaches (120, 170, 199), which also results in shorter in-hospital stay (195).

In general, the hybrid approach to close ventricular septal defects has demonstrated a high success rate, with an overall post-operative success rate around 95% or higher and complications in less than 5% of cases (229). For example, in a study of over 440 pediatric patients aged, on average, below 1 year, the successful closure rate was >96% with no reported deaths or major complications (201). Similarly, a successful closure rate of 96.6% was reported in a total of 320 pediatric patients aged between 1 and 3 years who were subjected to periventricular device closure (199). Even large muscular VSDs can be effectively closed via hybrid and percutaneous approaches (230). Within a 5-year period, the complete closure of the defect(s) occurred in 98.6% of the patients, according to a multicenter study (231). In a few cases, where the initial percutaneous closure was incomplete, the hybrid approach can be effective (220). However, certain types of heart defects, like Swiss cheese VSD, may require alternative approaches if the percutaneous device closure has failed (232).

Better psychological outcomes in patients subjected to the mini-invasive hybrid approach, compared to those treated via conventional surgical methods, are also a definitive advantage (233). The most important outcome of the hybrid approach is the minimization of cosmetic defects due to the diminished size of the surgical scar and the avoidance of cardiopulmonary bypass (227).

In addition to better clinical outcomes, the hybrid approach is considered a less costly approach because of its minimally invasive nature and shorter duration of the treatment. To our knowledge, there has been no systematic analysis of costs linked to traditional surgery and the hybrid approach. However, comparisons between open-heart surgery and the transcatheter (e.g., percutaneous) approach revealed that the latter is less expensive (209, 234). Because the hybrid approach does not require CPB and is free from CPB-associated complications and results in shorter in-hospital stay, one may conclude that the hybrid approach is less expensive compared to open-heart surgery. On the other hand, the need for a hybrid operating room and specialized personnel/equipment for transesophageal echocardiography increases the potential costs. Also, the actual costs in a certain country will likely depend on the overall development of the healthcare system itself and other fields closely linked to healthcare (e.g., the level of economic development, the existence of proper medical institutions, clinically approved protocols, etc.).

Among the limitations, failures in closure and rhythm disturbances are the main issues of the hybrid approach. It has been shown that the failure rate is somewhat higher for muscular VSDs than for perimembranous VSDs, with major complications of 5.3% vs. 1.2%, respectively (87). This study also reported a 1.6% occurrence rate of cAVB after the hybrid approach. Another study, in which complications were recorded in patients subjected to the hybrid approach (interventional VSD closure) between 1993 and 2015 in a single center, demonstrated an even lower complication rate, with only 0.7% for cAVB (100). Transcatheter closure of perimembranous VSDs may result in arrhythmias in approximately 25% cases, according to a study on 395 pediatric patients with a median age of 4 years (235). However, a similar cohort (320 patients) in another study did not demonstrate any rhythm disturbances or impairments of the conduction system (199). The brand of the closure device is unlikely to affect the outcomes. For example, a similar incidence of cAVB in ∼1-year-old patients was reported in a study using the Amplatzer Duct Occluder II (ADO II) and Konar MFO, with one case in each subgroup: 4.5% and 3.3% of the total number of patients, respectively (217). In another study on 77 pediatric patients with a median age of 4.3 years, there were no occurrences of permanent conduction block in the heart or other surgery-related complications if the patients were treated via ADO II or Konar MFO (204). In general, it can be concluded that the incidence of delayed cAVB is even lower in a hybrid setting compared to transcatheter percutaneous closure, despite the inevitable risk of this complication (191).

From the point of view of the authors, the long-term risk of cAVB is clear. Experience from our center shows that cAVB may develop in 1–3 years after implanting an occluder, although the number of such cases is low. The main reason may be the use of an improperly large occluder in small babies with VSDs. Also, out-of-hospital infections, like influenza, may elevate the risk of cAVB. Moreover, the most important factor is the type of occluder. We found that perimembranous occluders for VSD correction resulted in more frequent cAVB compared to the muscular type (unpublished data). It is likely that the effect is related to the size of the waist between the disks. It is therefore critically important not only to locate the position of the defect in the septum as accurately as possible but to assess the depth of the ridge of the septum. To conclude, a larger VSD, a smaller patient, and a perimembranous occluder altogether increase the risk of cAVB.

It should also be noted that the analysis for risk factor of complications in electrical propagation is difficult to perform, especially in heterogeneous cohorts of patients which may include patients with co-existing congenital heart defects and neurological syndromes. The comorbidities often have shared genetic basis (236), may involve impaired function/expression of ion channels, and develop due to improper maturation of both the heart and the brain (237, 238). This in turn determine the risk of rhythm disturbance and the block of electrical propagation because of direct involvement of activation/inactivation of cardiac ion channels to the process.

Damage to the aortic valve is another critical complication that may occur in transcatheter approaches, including a hybrid approach. Based on numerous hybrid treatments performed at our center, we concluded that the presence of at least 2 mm of aortic edge is critical to avoid damaging the semilunar aortic valve. Despite the existence of asymmetric occluders, which are specifically used for VSDs located close to the semilunar aortic valve, the presence of the aortic edge plays an important role in whether it is damaged. The damage may have various causes: (i) traumatic damage of the valvular flaps during manipulation, (ii) the stricture of the flaps during the expansion of the device, (iii) technical difficulties if non-adequate access to the defects is selected (the point of RV puncture), (iv) the experience of personnel that perform transesophageal control of implanting an occlude (139). There is still not enough data about the long-term outcomes of hybrid and transcatheter approaches in terms of potential atrial valve damage and conduction system complications (100, 103), so further studies are needed to elucidate these issues.

It should also be noted that the wider use of the hybrid approach is limited by the lack of standardized protocols, often resulting in individualized decisions based on clinicians’ experience, patient-specific symptoms, and anatomical findings (191, 239). Other issues include the need for further analysis of the long-term outcomes of hybrid approaches, particularly regarding the risk of cAVB (191), especially in patients with neuropsychological comorbidities (240). However, many large-scale comparative studies have confirmed that the hybrid approach is safer and more efficient compared to both traditional open-heart surgery and transcatheter percutaneous closure (195, 241). Further analysis of the effectiveness and safety of the hybrid approach in VSD closure is warranted as soon as more data become available from different cardiac surgery centers.

The annual (or preferably decade-based) rate of published papers concerning the hybrid approach remains high since the 2000s, and the available data show that this approach is effective for treating various VSDs, mostly perimembranous and muscular ones (77, 230). However, regional discrepancies in the use of this approach exist. For example, in the U.S., there are no FDA-approved occluders for transmyocardial (periventricular) closure of VSDs (while the Amplatzer Septal Occluder is an FDA-approved device for ASD closure). Thus, there are few reports from the U.S. on perventricular closure using the hybrid approach, instead mostly reporting extended corrective measures in adult patients who were previously subjected to VSD correction (242). Similarly, limited reports are available from other countries and regions, such as European countries (189, 243) and Japan (173); if the hybrid approach is used, it is mostly in adult patients. In contrast, the VSD occluder for periventricular closure in a hybrid setting under video-assisting guidance (via TTE) is widely used in China, especially for pediatric patients with CHDs, especially in recent years (77, 120, 170, 191, 241, 244, 245). Several reports and case series have shown that the hybrid approach is used in newborn, infant, and early childhood patients in Russia (199, 207, 246), Middle East (111), and Central East (43, 174). Despite the successful implementation of hybrid closure in most of the reported studies, it should be noted that the approach is most successful in carefully selected patients.

## 6 Prospects of using the hybrid approach to VSD closure

The hybrid approach to ventricular septal defect (VSD) closure, an effective strategy that bridges the gap between open-heart surgery and transcatheter interventions, continues to evolve (103, 194, 247). While current data demonstrate its feasibility and short-term safety in selected cases, future advancements and broader clinical integration depend on several key developments:

1) Refinement of patient selection criteria through evidence-based protocols will be crucial. Ongoing accumulation of clinical experience and real-world data will enable more precise stratification of patients based on anatomical characteristics, age, comorbidities, and risk factors, ensuring optimal outcomes and minimizing complications such as arrhythmias and device embolization. Also, the increasing interest in genotyping diagnosed CHDs (particularly VSDs) may lead to better outcomes of hybrid treatment in terms of better closure and diminished reopening, especially if preoperative genetic mapping is available (248).
2) Technological advancements in occluder design are expected to enhance procedural success and reduce device-related complications. Innovations aimed at improving anchoring mechanisms, biocompatibility, and retrievability may further increase the safety profile of the hybrid method, especially in infants and young children.
3) Integration of imaging technologies, including real-time 3D transesophageal echocardiography and intraoperative navigation systems, may improve accuracy in device placement, reduce intraoperative time, and further minimize myocardial trauma.
4) Developing standardized hybrid protocols and surgeon training programs will be vital for widespread adoption. Given the multidisciplinary nature of the procedure, requiring both surgical and interventional expertise, centers of excellence with dedicated hybrid teams will play a pivotal role in training and outcome benchmarking.
5) It is still not enough data that provide long-term outcomes of hybrid approach in closing VSD. Based on available data for middle-term observations, it is reasonably to conclude that hybrid approach is as safe as other types of surgery including conventional open-heart surgery (195, 241). However, this can not be simply extrapolated to longer periods of observation. In addition, the hybrid approach is not used extensively enough worldwide thus limiting both availability of late outcomes (complications), especially from large studies, and standardized protocols. Long-term follow-up studies and multicenter clinical trials are essential to assess durability, functional outcomes, and the incidence of late complications such as cAVB (103, 191). These data will provide the scientific foundation necessary for the formal incorporation of the hybrid approach into VSD management guidelines.

The progression and evolution of surgical treatment for CHDs (including VSDs) over time have led to a significant decline in overall mortality and the improvement of the quality of life of surviving patients (5, 60, 249). However, the reported post-operative mortality rates for surgical treatment of pooled types of CHDs remain significantly higher in low- and middle-income countries compared to those in high-income countries with well-developed healthcare programs (5, 10, 12, 26, 250–252). (Note that this applies only to countries/regions where the appropriate level of instrumental diagnostics and medical expertise is available, as a lack of diagnostic tools may result in low prevalence rates and CHD-related mortality rates.) Moreover, despite major advances in medical and surgical treatment of CHDs, physical, psychosocial, and neurodevelopmental outcomes remain a major concern. In a large cohort of patients with CHDs, aged between 19 and 38 years, about 40% reported at least one complication, such as troubles in hearing, vision, mobility, cognition, and self-care (253). Children with CHDs, particularly those undergoing repeated surgeries or prolonged hospitalizations, are at increased risk of cognitive and motor delays, as well as attention, learning, and behavioral difficulties (254, 255); this is partly related to the same genetic mutations that underlie CHD development (256).

It is now widely accepted that fetal echocardiography allows for early prenatal diagnosis, improving outcomes by facilitating delivery planning at specialized centers and enabling immediate postnatal intervention, including surgical treatment. The increased availability of early prenatal screening in many countries worldwide is now the main contributor to the dramatic increase in the diagnosis of fetal CHDs in recent decades. For instance, one study reported a significant rise from 6.2% in 1991 to 82.8% in 2021 (74). On average, 50%–60% of CHDs are now detected via fetal screening (75). However, while screening programs and neonatal care have enhanced survival rates, implementation remains inconsistent in many low- and middle-income countries (257).

As we have already mentioned in the previous section (Advantages and Limitations), the hybrid method for VSD correction is an elaborated approach that requires specific equipment and trained personnel. Therefore, it can be considered costly compared to some other interventions; however, taking into account lower risk for post-surgery complications (e.g., vs. conventional open-heart surgery) due to its mini-invasive nature it can be considered economically efficient. On the other hand, as we also mentioned above, the actual costs will depend on numerous factors and it is necessary to perform extended analysis to reveal whether the hybrid approach is more or less efficient in terms of expenses, resources, early outcomes, and late outcomes including the assessment of quality of life, especially in patients with severe comorbidities like neurological disorders.

These significant challenges encourage the use of more advanced but also less invasive surgical methods in countries with low reported mortality rates after CHD (VSD) correction and the implementation of further global and national efforts to develop pediatric healthcare systems and reduce the burden of CHDs (11). Among other advancements, the hybrid technique holds considerable promise as a less invasive yet highly effective modality for VSD closure. With continued innovation and rigorous clinical validation, it may eventually become a standard of care for selected patients.
